# Telbivudine and adefovir combination therapy for patients with chronic lamivudine-resistant hepatitis B virus infections

**DOI:** 10.1007/s00705-013-1786-4

**Published:** 2013-07-16

**Authors:** Ming-Tsung Lin, Yeh-Pin Chou, Tsung-Hui Hu, Hsien-Chung Yu, Yu-Chun Hsu, Ming-Chao Tsai, Po-Lin Tseng, Kuo-Chin Chang, Yi-Hao Yen, King-Wah Chiu

**Affiliations:** 1 Division of Hepato-Gastroenterology, Department of Internal Medicine, Kaohsiung Chang Gung Memorial Hospital, Chang Gung University College of Medicine, 123, Ta-Pei Road, Niao-Sung Dist., Kaohsiung, 833 Taiwan; 2 Division of Gastroenterology, Department of Medicine, Kaohsiung Veterans General Hospital, Kaohsiung, Taiwan; 3 Department of Gastroenterology, Changhua Christian Hospital, Changhua, Taiwan

## Abstract

We evaluated second-line salvage therapy with adefovir + telbivudine (group 1), adefovir followed by adefovir + telbivudine (group 2), or lamivudine + adefovir followed by adefovir + telbivudine (group 3) in hepatitis B patients with an inadequate virologic response to lamivudine treatment. Simple linear regression analysis showed that for each additional month of treatment, the most significant reduction in viral load occurred in group 1 (HBV DNA [Log_10_ IU/mL]: group 1, −0.149; group 2, -0.081; group 3, −0.123). Generalized estimating equation analysis revealed that compared to group 1, hepatitis B virus (HBV) DNA levels were 1.203 and 0.443 Log_10_ IU/mL higher in groups 2 and 3, respectively. Overall, a significant reduction in viral load (−0.060 Log_10_ IU/mL) was observed for each additional month of treatment. Adefovir + telbivudine treatment resulted in a significant reduction in HBV DNA levels. Moreover, telbivudine treatment resulted in a significant reduction in viral load (−0.050 Log_10_ IU/mL) compared to lamivudine treatment after the emergence of lamivudine resistance.

**Electronic supplementary material:**

The online version of this article (doi:10.1007/s00705-013-1786-4) contains supplementary material, which is available to authorized users.

## Introduction

Chronic hepatitis B virus (HBV) infection is a cause of significant mortality and morbidity worldwide. According to a WHO report published in 2008, two billion people were infected with the virus, and 350 million of these suffered from chronic HBV infection [1]. HBV DNA levels are the principal indicator of the extent of infection. Other indicators of infection include alanine transaminase (ALT) and hepatitis B e antigen (HBeAg); however, changes in these levels are dependent on the phase and extent of infection. HBV occurrence at birth or in the early stages of life is characterized by high levels of HBV DNA and HBeAg, but normal ALT levels. Indications for treatment depend on the presence or absence of HBeAg. Typically, HBeAg-positive patients with HBV DNA levels ≥ 20,000 IU/mL and elevated ALT levels of two times the upper limits of normal are considered for treatment [2, 3].

Lamivudine (LAM) is often considered to be the drug of choice for HBV patients due to its antiviral potency. However, a major disadvantage associated with conventional LAM monotherapy is the development of resistance [4, 5]. The polymerase gene encodes a DNA polymerase enzyme, which is needed for encapsidation of viral RNA into core particles and conversion of the pregenomic viral RNA into a negative strand of viral DNA. The mutations in the sequence of HBV DNA polymerase that confer drug resistance result in amino acid substitutions in the reverse transcriptase domain of the enzyme. The changes in the structure of the enzyme, in turn, are thought to inhibit binding of the drugs to their active sites [6].

LAM-induced resistance results from mutations in the HBV Pol gene, primarily rtM204I and rtM204V. Secondary mutations include rtL180M, and rtV173L [3, 7, 8]. It is estimated that more than 60 % of patients develop LAM resistance within four years of treatment [9]. The addition of or a switch to adefovir (ADV) or tenofovir (not available in Asia until early 2011) is recommended in patients with LAM-resistant HBV infections. However, some patients demonstrate inadequate responses with both ADV monotherapy and combination therapy. Recently, another L-nucleoside analogue, telbivudine (LdT), has demonstrated promising antiviral activity. A global trial suggested that LdT treatment resulted in better HBeAg reduction and seroconversion, lower treatment failure, and lower resistance and virologic breakthrough than LAM following two years of therapy [10, 11]. The lower resistance of LdT is attributed to the M204I mutation only, in comparison to the multiple LAM-induced mutations. Although LdT and ADV therapy is as effective as LAM and ADV therapy for patients with the M204I mutation, the lack of cross-resistance between ADV and LdT can also offer protection against ADV-induced resistance. Furthermore, the probability of new mutations is lowered, resulting in better viral suppression for a longer duration.

The main objective of this prospective study was to determine the efficacy of a combination treatment of LdT and ADV in patients with LAM-resistant HBV compared with either ADV monotherapy or LAM and ADV combination therapy. In addition, the ability of LdT to prevent ADV resistance in patients treated with a combination of both drugs was determined. HBV DNA levels were used for comparisons, as they are fairly accurate indicators of the extent of infection. With the results obtained from this study, we aimed to demonstrate that a combination of LdT and ADV treatment as opposed to the conventional therapy of ADV alone or LAM and ADV combination therapy for patients with LAM-resistant infections may be a better therapeutic option.

## Materials and methods

### Study population

Patients were recruited from the Chang Gung Memorial Hospital, Kaohsiung, Taiwan, in June 2007. The research was conducted in accordance with the Declaration of Helsinki and institutional standards and was granted ethical approval by the institute review board from Chang Gung Memorial Hospital (No. 100-2658B). Written informed consent for participation in the study was obtained from participants. All patients were subjected to second-line salvage therapy following virologic resistance to initial LAM therapy. All patients had virologic breakthrough (≥ 1 log _10_ after initial suppression of HBV DNA) during LAM treatment. The study subjects with LAM resistance were divided into three groups according to our inclusion criteria rather than using a randomized method. The study subjects with LAM-resistant HBV were divided into three groups. Group 1 included patients receiving ADV and LdT combination therapy after LAM resistance (n=11) after the study initiated in June 2007. These patients did not have LAM resistance until the initiation of this study. Group 2 included patients who received ADV monotherapy for LAM resistance before this study. They then received LdT and adefovir combination therapy after this study was initiated if they were found to show an inadequate response to ADV monotherapy (HBV DNA ≥ 200 IU/mL after 12 months of therapy) (n=9). Group 3 included patients who received a combination of LAM and ADV for LAM resistance before this study was initiated and then switched to LdT and ADV combination therapy after this study was initiated due to an inadequate virological response (HBV DNA ≥ 200 IU/mL after 6 months of therapy) (n=10). The drug information is a follows: telbivudine (Novartis Pharma Stein AG), 600 mg once daily; lamivudine (GlaxoSmithKline), 100 mg once daily; and adefovir (GlaxoSmithKline), 10 mg once daily.

### Follow-up

Patients were followed up every month with a clinical assessment as well as liver and renal biochemical tests. The serology of hepatitis B markers (including HBeAg and antibody to hepatitis B e antigen) was checked every six months for HBeAg-negative patients and every three months for HBeAg-positive patients. Serial HBV DNA levels were assessed at baseline (before either mono or combination ADV treatment) and every six months after ADV treatment. The YMDD motif region in the DNA polymerase gene was sequenced at baseline, at the time of biochemical and/or virologic breakthrough, or every six months. The end point of study was when HBV DNA became undetectable or when new resistance emerged after LdT plus adefovir therapy. The end date of the follow-up was 30 June 2012.

### Serological testing

The presence of hepatitis B surface antigen (HBsAg), HBeAg, and anti-HCV (hepatitis C virus) was assessed using commercial assay kits (HBsAg EIA, Abbott, Chicago, IL, USA; HBeAg EIA, Abbott; anti-HCV, EIA 3.0, Abbot). All of the patients were anti-HCV negative. The HBV DNA levels were quantified using a Cobas Amplicor HBV monitor kit (Roche Molecular Systems, Pleasanton, CA, USA) with a lower detection limit of 200 copies/mL. Dilution was performed if HBV DNA levels exceeded 106 copies/mL. Serum HBeAg levels were measured using a microparticle enzyme immunoassay (AxSYM; Abbott). The AxSYM assay results were based on the ratio of the sample (S) to the cutoff (Co) for each sample and control. HBeAg-positive and anti-HBe-positive findings were defined using S/Co ratios, in accordance with the manufacturer’s instructions (Abbott). Polymerase chain reaction and sequencing the HBV DNA polymerase gene mutations were done using nested PCR and direct sequencing as described previously [12]. The sensitivity of this method was 500 copies/mL.

### Statistics

Data were analyzed using simple regression analysis with the HBV DNA level as the dependent variable and treatment duration as the independent variable. Subsequently, semi-parametric generalized estimating equation (GEE) analysis was performed in order to determine the factors influencing the outcome of combination therapy as well as the outcomes of individual treatments and their duration. The HBV DNA level was the dependent variable, while the combination of drugs, usage of LdT, and treatment duration were independent variables. Pre-treatment HBV DNA levels were used as adjustment factors. A p-value of 0.05 (two-tailed) was considered statistically significant.

The generalized estimating equation is used to estimate the parameters of a generalized linear model with a possible unknown correlation between outcomes, especially for repeated measurements [13, 14]. In this study, a generalized linear model with a normal distribution and identity link function was used to assess the treatment effect at the HBV DNA level, and the generalized estimating equations (with working independence correlations and empirical robust SEs) was used to assess the change in the HBV DNA level within patients over time. The GEE takes into account the dependence between repeated observations from the same individual. The average effects in the population can be estimated.

## Results

### Patient demographics

The final analyses were performed on data collected from 30 patients (group 1, n = 11; group 2, n = 9; group 3, n = 10). The mean ages of the patients were 49, 57, and 43 years in groups 1, 2, and 3, respectively. There were 6 males and 5 females in group 1, 6 males and 3 females in group 2, and 6 males and 4 females in group 3. There was no significant age difference among three groups. The average duration of first-line LAM therapy is reported in Table 1. There was no significant difference among the three groups. The average durations of LAM, ADV, and LdT treatment after LAM resistance in each group are also reported in Table 1. Baseline HBV DNA levels (before ADV treatment) were not significantly different among the three groups (Table 1). There was also no significant difference in therapeutic duration among the three groups; however, a longer ADV and shorter LdT duration in Group 2 was noted. With regards to LAM resistance, the distribution of mutation points and patient numbers were as follows: rtM204I (5), rtM204V (3), rtM204V+rtL180M (2), and rtM204I+rtL180M (1) in group 1; rtM204I (3), rtM204V (3), rtM204V+rtL180M (2), and rtM204I+rtL180M (1) in group 2; rtM204I (3), rtM204I (3), rtM204V+rtL180M (3), and rtM204I+rtL180M (1) in group 3. There was no significant difference in the distribution of resistant strains between groups. LAM, ADV, and LdT were administered in doses of 100, 10, and 600 mg/day, respectively, and were adjusted according to the patients’ renal function.

**Table 1:** Characteristics of 30 chronic hepatitis B patients, including HBV DNA levels and duration of treatment

	Group 1 (N=11)	Group 2 (N=9)	Group 3 (N=10)	p-value
Baseline HBV DNA (Log10 IU/ml)	5.40 (±2.60)	6.72 (±1.43)	6.26 (±1.61)	0.421
LAM-experienced (months)	33.81 (±22.66)	29.77 (±19.09)	19.88 (±11.59)	0.265
AST	50.0 (±27.72)	62.1 (± 23.79)	46.66 (±25.53)	0.668
ALT	43.0 (± 30.17)	74.1 (±60.47)	49.66 (±23.09)	0.404
Treatment duration (months)				
LAM	–	–	11.26 (±4.93)	–
ADV	23.95 (±7.66)	44.36 (±18.10)	34.83 (±9.69)	0.006*
LdT	23.63 (±7.41)	20.28 (±5.44)	22.59 (±7.47)	0.649

Baseline DNA indicates the DNA levels before treatment with ADV

### HBV DNA levels

The HBV DNA levels of 30 chronic hepatitis B patients were analyzed. Before the second-line salvage therapy, all of the patients had received lamivudine therapy, and resistance and virologic breakthrough had occurred. The average HBV DNA concentration was 5.40 (Log10 IU/ml) in group 1, 6.72 (Log10 IU/ml) in group 2, and 6.26 (Log10 IU/ml) in group 3. The durations of prior LAM treatment were not significantly different, as indicated.

### Linear regression analysis

**Fig. 1:**
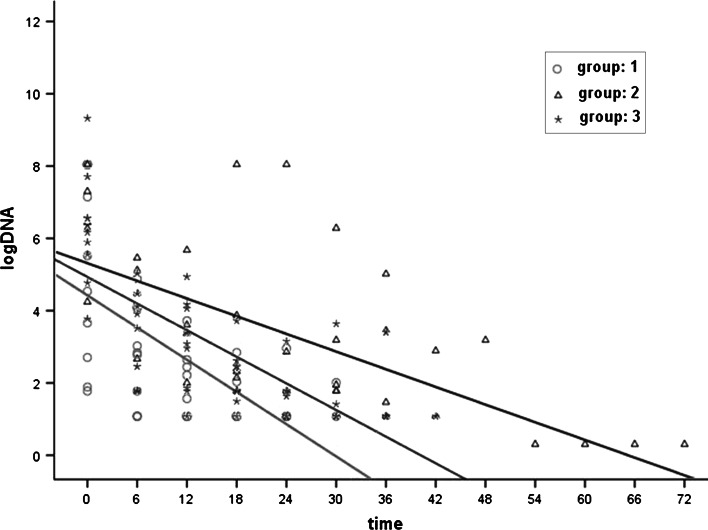
Linear regression analysis of treatment duration and reduction in HBV DNA levels. All three groups showed a reduction in HBV DNA concentrations with increasing time of treatment. Of the three groups, the reduction in group 1 was the most prominent, with a 0.149 (Log10 IU/ml) reduction in HBV DNA concentration for each month of prolonged treatment. However, the linear regression was not significantly different between the three groups, as indicated by the low R-Sq values (0.361, 0.406, and 0.514, respectively)

To evaluate the correlation of different treatments with reductions in HBV DNA levels, we used HBV DNA (Log10 IU/ml) as the dependent variable and treatment duration as the independent variable. Table 2 illustrates the results of simple linear regression, and all three groups showed a decrease in viral DNA levels within the treatment period (p < 0.001). Group 1 had the most potent reduction of 0.149 (Log10 IU/ml) for each month of treatment. However, the linear regression was not significantly different between the three groups, as indicated by low R-Sq values (0.361, 0.406, 0.514, respectively, Figure 1 and Table 2).

**Table 2:** Linear regression analysis of treatment duration and HBV DNA reduction

	B	SEb	T	P-value	R-square	Adj R-Sq
Group 1						
Time (months)	−0.149	0.029	−5.087	<0.001	0.376	0.361
Group 2						
Time (months)	−0.081	0.015	−5.577	<0.001	0.420	0.406
Group 3						
Time (months)	−0.123	0.016	−7.559	<0.001	0.524	0.514

β (beta), regression coefficient; SEb, standard error of beta; T, *t* statistic

### GEE analysis

To evaluate the correlation of different treatments with reductions in HBV DNA levels using more-accurate adjustments, we performed GEE analysis. The dependent variable was the HBV DNA level (Log10 IU/ml), and the independent variables were (1) combination of drugs, (2) usage of LdT, and (3) treatment duration for each drug. The adjustment factor was HBV DNA (Log10 IU/ml) before ADV treatment. Overall, a reduction of 0.06 (Log10 IU/ml) in HBV DNA concentration (p < 0.001, Table 3) was found for every month of prolonged treatment. Analysis of the different treatments and their respective durations was subsequently performed. Compared to group 1, group 2 showed 1.203 (Log10 IU/ml) higher HBV DNA concentrations (p < 0.001), and Group 3 showed 0.443 (Log10 IU/ml) higher HBV DNA concentrations (p = 0.123, Table 3) after treatment. Group 1 patients exhibited a better virologic reduction than group 2 or group 3 patients. As both groups 2 and 3 involved two kinds of subsequent treatment, we evaluated the correlation of each treatment with the reduction in HBV DNA when compared with ADV-treatment alone (before adding LdT in group 2). The results showed that ADV + LdT (group 1) treatment resulted in a better reduction of 1.593 (Log10 IU/ml) in HBV DNA concentrations than ADV monotherapy (group 2) (p < 0.001). ADV + LAM (group 3) treatment also showed a borderline better reduction of 0.761 (Log10 IU/ml) in HBV DNA concentrations compared to ADV monotherapy (p = 0.066) (Table 4). These results are consistent with a previous report that suggested that combination therapy may provide a better HBV DNA reduction than ADV alone. Furthermore, after adjusting for the length of treatment with the three drugs, LdT treatment was found to yield the most powerful reduction in HBV DNA concentration of 0.050 (Log10 IU/ml) for each month after LAM resistance (p = 0.004). ADV also contributed to a reduction of HBV DNA concentration of 0.025 (Log10 IU/ml) for each month of treatment (p = 0.001). However, the contribution of LAM in the reduction of HBV DNA was no longer significant if LAM resistance developed (p = 0.50) (Table 5). These results indicate that LdT played a more important role in HBV DNA reduction given that ADV + LdT treatment showed the most significant reduction. Furthermore, the mean value of the log_10_ HBV DNA level for group 2 and group 3 before LdT treatment is 3.58 (SD=1.41), and that is after 1.98 (SD=1.14) LdT treatment (p-value <0.0001). This means the HBV DNA levels were reduced after combination treatment with LdT. After taking into account the dependence of repeated observation, the average reduction of log_10_ HBV DNA levels is -1.18 (p-value=0.0091).

**Table 3:** Generalized estimating equation analysis of different combination therapies compared to group 1

	B	Std. error	95 % Wald C.I.		p-value
			Lower	Upper	
Group 1	0	–	–	–	–
Group 2	1.203	0.330	0.548	1.857	<0.001
Group 3	0.443	0.285	−0.123	1.009	0.123
Time (months)	−0.060	0.005	−0.069	−0.051	<0.001
Baseline DNA (Log10 IU/ML)	0.233	0.067	0.101	0.366	0.001

**Table 4:** Generalized estimating equation analysis of different treatments compared to ADV treatment alone (before adding on LdT)

	B	Std. Error	95 % Wald C.I.		p-value
			Lower	Upper	
ADV + LdT	−1.593	0.282	−2.161	−1.026	<0.001
ADV + LAM	−0.761	0.407	-1.573	0.511	0.066
ADV	0	–	–	–	–
Time (months)	−0.075	0.015	−0.105	−0.045	<0.001
Before DNA (Log10 IU/ml)	0.189	0.063	0.064	0.314	0.003

**Table 5:** Generalized estimating equation analysis of different agents and treatment duration compared with ADV treatment alone and adjusted for duration of treatment with each drug

	B	Std. error	95 % Wald C.I.		p-value
			Lower	Upper	
ADV+LDT	−1.510	0.377	−2.271	−0.749	<0.001
ADV+LAM	−0.441	0.537	−1.510	0.627	0.414
ADV	0	–	–	–	–
Treatment duration (months) LAM	−0.015	0.021	-0.057	0.028	0.500
ADV	−0.025	0.007	−0.040	−0.010	0.001
LdT	−0.050	0.017	−0.084	−0.016	0.004
Baseline DNA (Log10 IU/ml)	0.258	0.063	0.132	0.384	<0.001

### Genotypic resistance to ADV and LMV

Genotypic resistance to ADV was investigated for all patients at baseline and every six months after ADV-based treatment. Only one patient in group 1 had the rtA181T mutation at baseline (due to previous LAM treatment), and one patient in group 2 had the rtA181V and rtN236T mutations before LdT add-on therapy (due to ADV monotherapy) (Table 6). Table 6 shows the time course of the virologic response and ADV resistance profile after ADV-based treatment in these 30 patients. Despite the emergence of these two mutations, serum HBV DNA levels continued to decline progressively in all 30 patients, becoming undetectable in 9 of 11 (81 %) patients in group 1, 5 of 9 (55 %) patients in group 2, and 7 of 10 (70 %) patients in group 3 after at least two years of therapy. The rates of *de novo* genotypic resistance to rtA181T and rtN236T after LdT-ADV combination therapy were both 0 % at the end of the follow-up period. By the end of the study, both the rtA181T mutation in group 1 and the rtA181V mutation in group 2 had disappeared after adding LdT therapy for 12 and 18 months, respectively.

**Table 6:** Virological response and ADV resistance after treatment of 30 patients with LAM-resistant HBV infections for two to three years with ADV + LdT

	Group		
Virological response (follow-up 104W-208W)	ADV+LDT (n=11)	ADV→ADV+LDT (n=9)	LAM+ADV→ ADV+LDT (n=10)
HBV-DNA undetectable	9/11 (81 %)	5/9 (55 %)	7/10 (70 %)
HBeAg loss	3/6 (50 %)	1/3 (33 %)	2/5 (40 %)
Virologic breakthrough	0 (0 %)	1/9 (11 %)	1/10 (10 %)
Genotypic ADV-R	0 (0 %)	1/9 (11 %)	0 (0 %)
rtA181T	1/11 (20 %)	0 (0 %)	0 (0 %)

### ALT and serologic response

Twenty-four patients (80 %) with raised baseline levels of ALT showed ALT normalization during treatment, at rates of 29/30 (96 %), 100 %, and 100 % after 1, 2, and 3 years, respectively. Among the 6 patients with normal ALT levels at baseline, none had an elevated ALT level during treatment. Overall, one patient in Group 2 had a virologic breakthrough during ADV monotherapy. Six of 15 patients (40 %) lost HBeAg, and 3 (20 %) seroconverted to antibody to hepatitis B e antigen after ADV-based treatment. None of these patients cleared serum hepatitis B surface antigen with antiviral therapy.

### Safety

No significant adverse events were reported during the course of the study. Most patients had normal renal function during treatment. ALT and creatinine kinase levels remained under control in all patients.

## Discussion

The selection of an appropriate treatment strategy is critical for patients with chronic hepatitis B. The management of patients with HBV infection should involve treatment that consistently reduces viral load and prevents the development of mutations that result in drug resistance. Long-term LAM monotherapy is known to favor an increase in mutations by 20 % within the first year and by 70 % in the first five years of therapy [3, 15–17]. Mutated strains of HBV are known to replicate more rapidly with antiviral treatment. These strains are also diffuse and expand throughout the hepatocyte parenchyma, eventually invading the peripheral blood [18]. The development of drug resistance also has clinical implications such as decomposition, rapid progression to liver cirrhosis, and hepatocellular carcinoma [19–21]. It is recommended that a switch to ADV therapy be made as early as possible. However, this strategy does not prevent the development of new mutations, and patients often develop resistance to ADV therapy without an adequate reduction in HBV DNA levels. Studies evaluating the long-term risk of genotypic resistance to ADV in patients already resistant to LAM indicate that over a quarter of the patients develop ADV resistance within 1 to 2 years of ADV monotherapy [22–25]. This indicates that switching to ADV monotherapy is not an optimal option for patients with LAM-resistant strains. Recent studies have indicated that add-on ADV treatment (such as a LAM + ADV combination) for patients with LAM-resistant infections provides better viral suppression and helps to prevent additional ADV resistance [26, 27]. However, in our experience, some patients still have an inadequate response to either ADV monotherapy or LAM + ADV combination therapy, suggesting that there should be another option of combination therapy for clinical practice. Hence, we hypothesized that the use of LdT instead of LAM may be a better option for ADV-based combination therapy.

Both LdT and LAM are L-nucleoside analogues. Global trials of LdT have demonstrated a better virologic suppression, better HBeAg loss and seroconversion, less treatment failure, and less viral resistance and virologic breakthrough than is observed with LAM after 2 years of therapy [10, 11]. LdT has fewer mutation points than LAM and only induces the YIDD mutation (rtM204I), in contrast to the YVDD + YIDD (rtM204V or rtM204I) mutations induced by LAM [10, 11]. Therefore, we hypothesized that LdT + ADV may be a better way or another option to treat patients with LAM-resistant HBV infections than LAM + ADV. In patients with the YIDD mutation, the viral suppression of LdT + ADV should not be inferior to LAM + ADV. More importantly, LdT + ADV should theoretically not induce a new additional YVDD mutation (which may occur with LAM + ADV). In addition, in patients with the YVDD mutation, LdT + ADV treatment may result in better viral suppression than treatment with LAM + ADV [28]. The risk of inducing new YIDD mutations is theoretically equal between LdT + ADV and LAM + ADV therapy. We hypothesized that the protection of additional ADV resistance would also be equal because of a lack of cross-resistance between LdT and ADV. Entecavir (ETV) monotherapy (1 mg) has been reported to have the risk of inducing additional mutations in patients with LAM-resistant infections because ETV has cross-resistance with LAM (rtM204M/I) [29]. There are no published data on ETV + ADV therapy for patients with LAM-resistant infections; however, there may be a risk of inducing new additional mutations in long-term ETV + ADV therapy (including the positions rt184, rt202, and rt250). In contrast, there is no additional risk for LdT + ADV therapy, since only rtM204I and A181T have been found in global trials [8]. More importantly, a single rtA181T mutation did not induce any virologic breakthrough in that report. Salvage therapy with monotherapy (even with tenofovir: TDF) is not recommended for patients with LAM-resistant HBV infections under the present guidelines [30], and combination therapy is the mainstay (for example, truvada: TDF + emtricitabine [FTC]). TDF is a better substitute for ADV; however, TDF was not available in Taiwan or other parts of Asia until early 2011. Even though TDF-based therapy is used, LdT may still be a better choice in TDF-based combination therapy rather than FTC, since FTC has also been reported to induce both rtM204V and rtM204I mutations. Taken together, we believe that LdT + ADV combination therapy may be a better regimen at present than LAM + ADV therapy for patients with lamivudine-resistant strains, in Taiwan or anywhere where TDF is not available.

This prospective study was conducted to determine the efficacy of combination therapy with ADV and LdT as second-line salvage therapy for patients with LAM-resistant HBV infections. A positive correlation exists between the HBV DNA levels and the cumulative occurrence of hepatocellular carcinoma [31]. Hence, the regulation of HBV DNA levels within an acceptable limit is an essential goal of HBV therapy. We observed that the most prominent reduction in HBV DNA levels was in group 1, in which patients received ADV and LdT. Furthermore, after adjusting for all independent variables such as combination of drugs, usage of LdT, and treatment duration of each drug, LdT treatment showed a statistically significant decrease in HBV DNA concentration for each month of prolonged treatment. ADV also contributed to a reduction of HBV DNA for each month of treatment; however, the contribution of LAM in the reduction of HBV DNA concentrations was no longer significant if LAM resistance developed (p = 0.50) (Table 5). No adverse events were reported, and renal function was normal in all patients following LdT treatment.

Other antivirals such as entecavir carry the risk of inducing secondary mutations when administered in combination with ADV as long-term therapy in patients with LAM-resistant strains. However, there was no evidence of new mutations leading to ADV resistance following administration of ADV and LdT as combination treatment in this study. This result could be of considerable consequence for HBV therapy, as mutated strains replicate more aggressively in the presence of antivirals as a part of their survival and escape strategy [19, 32].

The main objective of this prospective study was to determine the efficacy of a combination treatment of LdT and ADV in patients with LAM-resistant HBV infections compared with either ADV monotherapy or LAM and ADV combination therapy. We used a prospective repeated measurement design to evaluate the efficacy of HBV viral reduction. Patients were followed up every month with a clinical assessment as well as liver and renal biochemical tests. In addition, hepatitis B markers were checked every six or three months for HBeAg-negative and positive patients, respectively. Importantly, since the HBV DNA levels change over time and the two measurements of HBV DNA levels in the same patient are interdependent, repeated measures analysis was performed using a generalized estimating equations (GEEs) method to adjust for this. (Tables 3, 4, 5). This method takes into account the dependence between repeated observations within same individual. The main advantage of GEE resides in the unbiased estimation of the population-averaged reduction effect on HBV DNA levels despite possible misspecification of the correlation structure.

In conclusion, in patients with LAM-resistant HBV infections, combined ADV and LdT therapy reduced the risk of genotypic resistance to ADV, preventing virologic and clinical breakthrough during a 2- to 3-year period. Although the patient numbers are relatively small in this study, the data provide vital insights into the administration of LdT in countering the drawbacks of existing HBV treatments. These results suggest a novel treatment approach that warrants further confirmatory analysis in a randomized controlled trial.

## Electronic supplementary material

Below is the link to the electronic supplementary material.

Supplementary material 1 (DOCX 86 kb)

## Abbreviations

LAM: Lamivudine
ADV: Adefovir
LdT: Telbivudine
HBV: Hepatitis B virus
ALT: Alanine transaminase
RT: Reverse transcriptase
HBsAg: Hepatitis B surface antigen
HBeAg: Hepatitis e antigen
GEE: Generalized estimating equation
TDF: Tenofovir
FTC: Emtricitabine
ETV: Entecavir
YMDD: Tyrosine-methionine-aspartate-aspartate
YVDD: Tyrosine-valine-aspartate-aspartate
YIDD: Tyrosine-isoleucine-aspartate-aspartate
