# Supplementary material for: Telbivudine and adefovir combination therapy for patients with chronic lamivudine-resistant hepatitis B virus infections
Source: Arch Virol. 2013 Jul 16;159(1):29–37. doi: 10.1007/s00705-013-1786-4 (PMC3888503; doi:10.1007/s00705-013-1786-4)
Supplement: Supplementary file 1 — Supplementary material 1 (DOCX 86 kb) [file 705_2013_1786_MOESM1_ESM.docx]

Supplemental table 1 Baseline characteristics of 91 chronic hepatitis B patients, including HBV DNA, gender and LAM duration of treatment.

Baseline characteristic

|  |  |  | |  | | |  | |
| --- | --- | --- | --- | --- | --- | --- | --- | --- |
|  | ADV (N=28) | | LAM+ADV(N=52) | | ADV+LDT(N=11) | P-value | |  |
| Baseline DNA, MeanSD (Log) 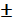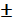 | 6.09 1.48 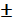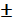 | | 5.191.57 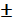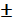 | | 5.402.60 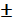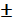 | 0.076 | |  |
| Gender, male | 24 (85.71%) | | 41(78.85%) | | 6(54.55%) | 0.104 | |  |
| LAM-experienced (month) | 22.05±10.38 | | 30.75±18.6 | | 33.81(±22.66) | 0.098 | |  |

Supplemental figure 1

**Linear regression of treatment duration and reduction in HBV DNA.**

All three groups showed reductions in HBV DNA concentrations with increasing time of treatment. Of the three groups, the reduction in Group of ADV+LdT was the most prominent, with a 0.149 (Log10 IU/ml) reduction in HBV DNA concentration for every month of prolonged treatment. (Supplemental table 2)


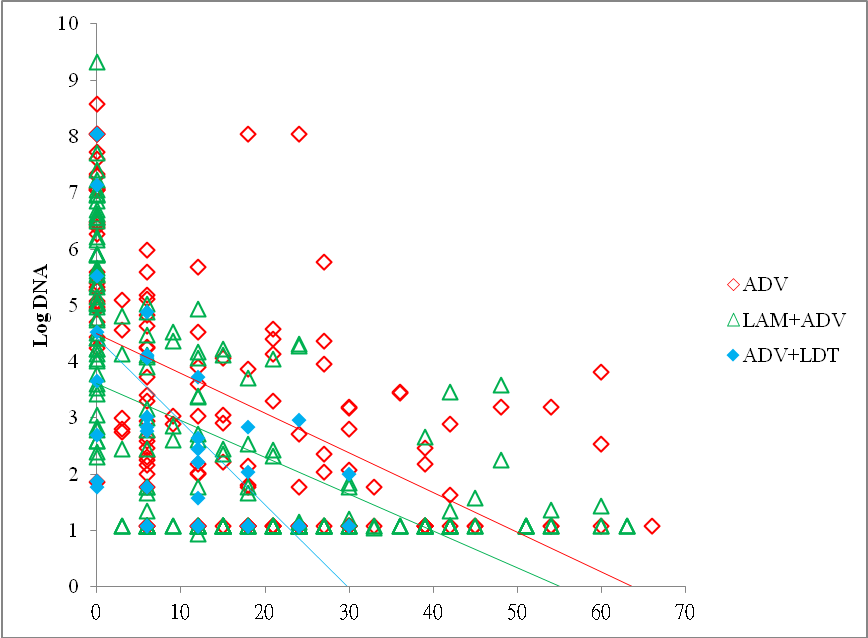


Supplemental table 2

Simple linear regression model

|  | B | SEb | P-value |
| --- | --- | --- | --- |
| ADV+LdT |  |  |  |
| Time (month) | -0.149 | 0.0285 | <0.0001 |
| ADV |  |  |  |
| Time (month) | -0.070 | 0.0092 | <0.0001 |
| LAM+ADV |  |  |  |
| Time (month) | -0.066 | 0.0059 | <0.0001 |

Supplemental table 3. Generalized estimating equation analysis of different treatments compared to ADV treatment alone. The results showed that ADV + LdT treatment had a better reduction of HBV DNA concentrations (B = -0.572) than ADV monotherapy (p = 0.036). ADV + LAM treatment showed a borderline better reduction of HBV DNA concentrations (B= -0.320) compared to ADV monotherapy, but without statistical significance (p = 0.12).

|  |  |  | | 95% Wald C.I. | | | | |  | |
| --- | --- | --- | --- | --- | --- | --- | --- | --- | --- | --- |
|  | B | | | | Std. Error | Lower | Upper | p-value | |  |
| ADV + LdT | -0.579 | | 0.277 | | | -1.122 | -0.036 | 0.0366 | |  |
| ADV + LAM | -0.320 | | 0.207 | | | -0.725 | 0.085 | 0.1212 | |  |
| ADV | 0 | | -- | | | -- | -- | -- | |  |
| Time (month) | -0.074 | | 0.005 | | | -0.084 | -0.063 | <0.0001 | |  |
| Before DNA (Log10 IU/ml) | 0.428 | | 0.047 | | | 0.335 | 0.521 | <0.0001 | |  |
